# The Cost Effectiveness of Immunoglobulin vs. Hematopoietic Stem Cell Transplantation for CIDP

**DOI:** 10.3389/fneur.2021.645263

**Published:** 2021-03-22

**Authors:** Richard K. Burt, Paul Tappenden, Roumen Balabanov, Xiaoqiang Han, Kathleen Quigley, John A. Snowden, Basil Sharrack

**Affiliations:** ^1^Division of Immunotherapy, Department of Medicine, Northwestern University Feinberg School of Medicine, Chicago, IL, United States; ^2^Health Economics and Decision Science, School of Health and Related Research, University of Sheffield, Sheffield, United Kingdom; ^3^Department of Neurology, Northwestern University Feinberg School of Medicine, Chicago, IL, United States; ^4^Department of Haematology, Sheffield Teaching Hospitals NHS Foundation Trust, University of Sheffield, Sheffield, United Kingdom; ^5^Academic Department of Neuroscience and Sheffield, NIHR Translational Neuroscience BRC, Sheffield Teaching Hospitals NHS Foundation Trust, University of Sheffield, Sheffield, United Kingdom

**Keywords:** health economics, immunoglobulin, hematopoietic stem cell transplantation, CIDP, cost

## Abstract

**Background:** Intravenous immunoglobulin (IVIG) is effective as standard first line therapy for chronic inflammatory demyelinating polyradiculoneuropathy (CIDP), but some patients remain dependent on its long-term use. Recently, we have reported that autologous non-myeloablative hematopoietic stem cell transplantation (HSCT) is an effective second line therapy for CIDP.

**Objectives:** To compare the cost of chronic IVIG vs. autologous HSCT (a one-time therapy), we collected data on patients with CIDP undergoing HSCT between 2017 and 2019. This was compared with published literature on the costs and efficacy defined by the Inflammatory Neuropathy Cause And Treatment (INCAT) disability score, Medical Research Council (MRC) sum score, hand grip strength, and SF-36 quality of life (QOL) for CIDP.

**Methods:** Between 2017 and 2019, nineteen patients with chronic CIDP (mean disease treatment duration prior to HSCT of 6 years) underwent autologous HSCT with mean cost of $108,577 per patient (range $56,327–277,119, standard deviation $53,092). After HSCT, 80% of patients remain IVIG and immune treatment free for up to 5 years. In comparison, published cost of IVIG treatment in the USA for an average CIDP patient exceeds $136,000 per year. Despite remaining treatment free, HSCT demonstrated greater improvement in efficacy compared to immunoglobulins.

**Recommendations:** Given the long-term treatment-free remission and better outcome measurements, autologous HSCT is more cost effective than long-term IVIG treatment in patients with chronic CIDP. However, costs will depend on patient selection, the HSCT regimen, and regional variations. Further analysis of the health economics, i.e., cost/outcome ratio, of HSCT as therapy for chronically IVIG dependent CIDP is warranted.

## Introduction

CIDP is a demyelination disease of the peripheral nervous system that may pursue a monophasic course of more than 2 months before remitting, follow a relapsing-remitting course, or maintain a chronic progressive course. In its classical presentation, CIDP is characterized by bilateral symmetrical motor and sensory deficits that occur in both the proximal and distal peripheral nervous system and by diminished tendon reflexes (1, 2). “Atypical” CIDP variants are: multifocal acquired demyelinating sensory and motor (MADSAM) (also known as Lewis-Sumner Syndrome), distal acquired demyelinating symmetric (DADS), and pure motor or pure sensory polyneuropathy. CIDP is a rare disease, affecting 1.6–10.3/100,000 adults and is one of the few peripheral neuropathies that responds to immune based therapies.

First line therapies are corticosteroids, IVIG (3, 4), subcutaneous immunoglobulin (SCIg) (5), and plasmapheresis (PLEX) (6). However, some patients fail to respond to first-line treatments (7) or respond but become dependent on their long-term use (8). Depending on the literature, 25–60% of patients can be weaned off their treatment (9, 10) while ~15–30% remain refractory to and/or dependent on treatments (11). IVIG and SCIg are expensive, especially when required for chronic long-term treatment, restrict quality of life, and may have significant side effects including anaphylaxis, aseptic meningitis, and maintenance of and complications from chronic intravenous access. The financial burden of chronic IVIG or losing employment and subsequent loss of employment insurance benefits can prevent or be a significant hindrance to continuation of expensive long-term treatment with IVIG (12–15).

We have previously demonstrated that non-myeloablative autologous HSCT is effective in inducing a drug free clinical remission in patients with CIDP who failed or are dependent on either IVIG and or PLEX (16). Following HSCT up to 80% of such patients became free of all immune based treatments including IVIG over a follow-up interval of 5 years (16). Further, treated patients had significant improvements in INCAT disability score, MRC sum score, hand grip strength, SF-36 QOL scores, and in nerve conduction studies (NCS) including nerve conduction velocity (NCV) and compound motor action potentials (CMAPs) that persisted for the 5 years of follow-up observation (16). Costs and re-imbursement for HSCT were not captured during that study. We, therefore, undertook an analysis of cost for autologous HSCT between 2017 and 2019 compared to published literature for those on IVIG treatments.

## Methods

### Setting and Design

Enrollment in the CIDP HSCT trial was completed in 2016 and published in June 2020 (16). As the costs of HSCT in that study could not be retrospectively recovered, we analyzed the costs of HSCT between January 2017 and January 2019 for patients treated in an identical manner using the same protocol and same eligibility and standard of care guidelines. Costs were compared to recent published literature on costs for treating CIDP with IVIG.

### Eligibility

As described previously (16), eligible patients had definitive CIDP according to the European Federation Neurologic Societies/Peripheral Nerve Society (EFNS/PNS) criteria (1) and dependence on or failure of at least two of the following three standard treatments: corticosteroids, IVIG, or PLEX (16).

### Autologous Hematopoietic Stem Cell Transplantation

As previously described (16), peripheral blood hematopoietic stem cells were collected using cyclophosphamide (2 g/m^2^) and filgrastim (5–10 mcg/kg/day), cryopreserved, thawed, and refused without manipulation after receiving a conditioning regimen of cyclophosphamide (200 mg/kg), rabbit antithymocyte globulin (rATG) (5.5 mg/kg), and rituximab 1,000 mg. Supportive care guidelines were previously reported (16).

### Hematopoietic Stem Cell Transplantation Costs

Except for physician billing, all hospital transplant related costs were counted including: blood draws, laboratory tests, imaging studies, peripheral blood stem cell collection and reinfusion, nursing care, packed red blood cell and platelet transfusions, room charges, and oral or intravenous medications including electrolyte infusions, diuretics, antibiotics, anti-emetics, and fluids.

HSCT costs are reported as direct, overhead, and total costs. Direct costs are costs of patient treatment and care. Overhead costs are the management and maintenance expenses required to run a hospital. The sum of direct and overhead costs are total costs.

### IVIG Costs

We searched PubMed for all manuscripts (excluding abstracts and non-peer-reviewed materials) under the terms “CIDP,” “costs,” and “immunoglobulin” published over roughly the same time interval (years 2014–2020) as the data collected on costs for patients undergoing hematopoietic stem cell transplantation, i.e., years 2017–2019.

### Outcomes

In order to assess cost effectiveness, we searched PubMed under the terms “CIDP,” “IVIG,” and “hematopoietic stem cell transplantation” for clinical outcomes between treatment with IVIG vs. HSCT. Search parameters were limited to English and the decade of 2014–2020. The intent of this analysis is to provide an initial platform to support a more in depth future analysis of the healthcare economics of HSCT vs. IVIG for chronic progressive CIDP. Clinical outcomes included were INCAT disability score, MRC sum score, hand grip strength, QOL, or NCS including NCV or CMAPs.

### Statistics

Due to absence of prospective comparison data available in the literature, costs are based on single treatment studies and could not be based on randomized comparisons between IVIG and HSCT. We calculated the mean HSCT cost in the entire cohort and for each patient in order to represent deviation of each patient from the mean. Similarly, outcome measures could not be obtained from randomized trials, but are included as a rough estimate to gauge cost effectiveness for patients who are maintained on IVIG vs. those who undergo a one-time treatment of HSCT with subsequent discontinuation of all immune therapies.

## Results

### Costs of HSCT

**Figure 1:**
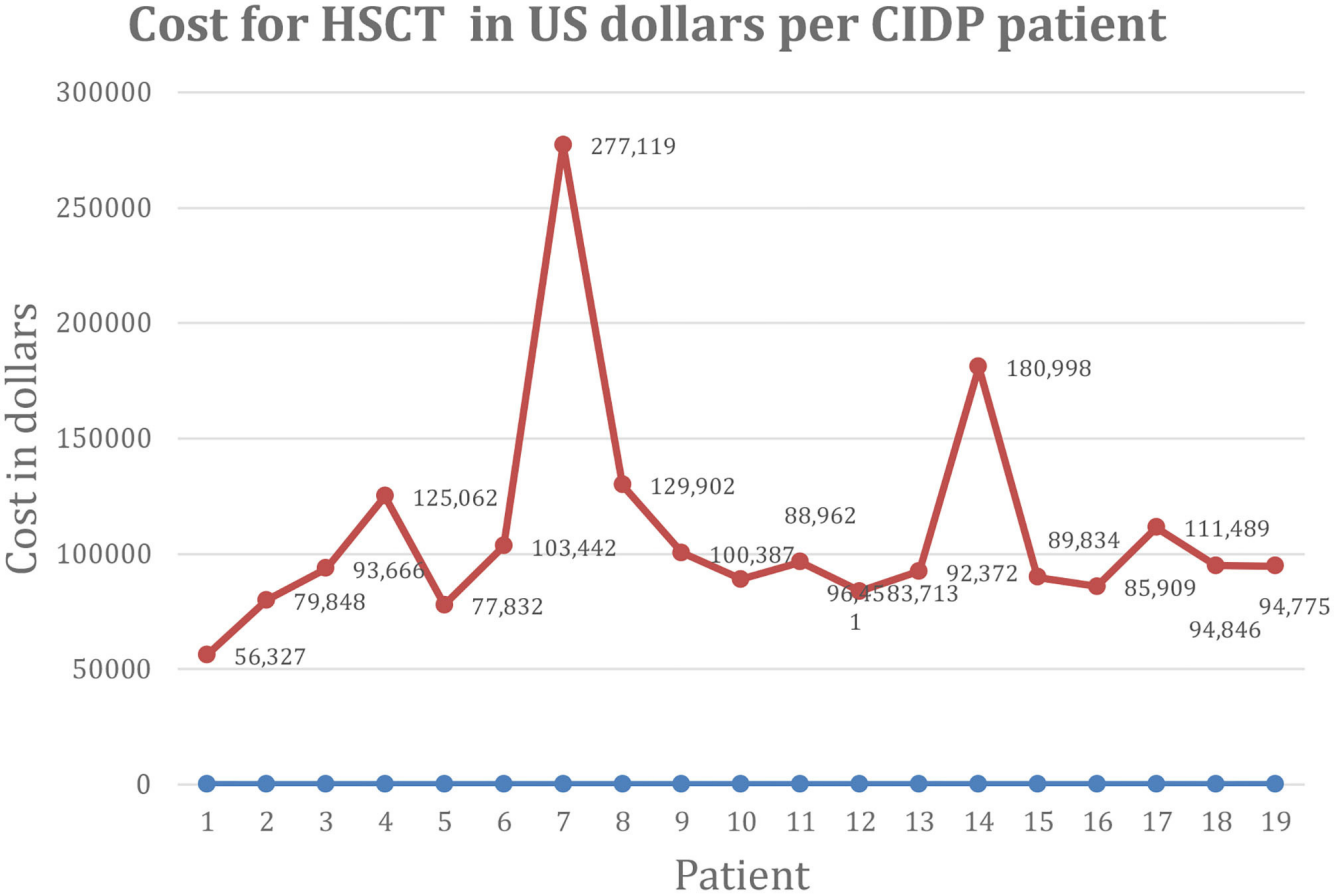
Cost of HSCT in US dollars per CIDP patient. CIDP, chronic inflammatory demyelinating polyradiculoneuropathy; HSCT, hematopoietic stem cell transplantation; US, United States.

We found no previously published manuscripts on the cost of autologous HSCT for CIDP. The mean direct, overhead, and total costs of HSCT were $62,131 (range $32,942–168,097), $46,446 (range $21,386–62,555), and $108,577 (range $56,327–277,119), respectively (Figure 1). The mean revenue collected and net profit was $140,812 (range $72,702–278,722) and $31,531 (range $68,673 to –30,740) per patient, respectively. As shown in Figure 1, there was one outlier with high HSCT costs of $277,119 that drove the mean total costs from $99,218 (mean for 18 patients) to $108,576 (mean for 19 patients), and a second low outlier in net profit, i.e., a loss of $30,740, due to fixed below cost medicare reimbursement rules.

The outlier with high cost was a patient receiving daily IVIG 5 times a week alternating with 4 times a week every other week. While in all other patients, IVIG was discontinued on day of hospital admission, this patient had a history of sudden neurologic deterioration requiring intensive care unit admission when a single dose of IVIG was delayed. For that reason, the patient was maintained on IVIG during HSCT and those costs were included in his HSCT billing. The patient was losing intravenous access from chronic IVIG administration and had a broken and retained intravascular central line whose removal further inflated costs.

### Cost of IVIG for CIDP

Review of the literature, excluding abstracts, meeting presentations, non-peer reviewed and Guillain-Barré syndrome related papers, revealed five peer reviewed publications from the USA (*n* = 2) (17, 18), UK (*n* = 1) (12), France (*n* = 1) (19), and Germany (*n* = 1) (20) related to health care costs of CIDP between 2014 and 2020 (Table 1). The costs of CIDP treatment with IVIG was £49,430 in the UK (19) and €45,332 in Germany (20) (Table 1). The French study reported that the cost for at home infusion of IVIG was €48,189/year vs. €91,798/year for in hospital infusions (19). In 2014, the annual cost for IVIG in the USA was on average $108,016 per patient or $9,720 per infusion (17). In 2018, the costs of treating CIDP in the USA was $136,892 (18). In all CIDP studies the main patient care cost, 51–67%, was due to IVIG (Table 1).

**Table 1:** Examples of variability in cost of care for IVIG treatments vs. HSCT for CIDP from one center.

**Study/Country/Year (reference)**	**Cost per patient**	**Comment**
**IVIG treatments**		
Mengel/Germany/2018 (20)	€45,332 per year	67% of cost relates to cost of IVIG given in hospital
Madhi-Rogers/UK/2014 (12)	£49,430 per year	62% of cost relates to cost of IVIG given in hospital
Le Masson/France/2017 (19)	€48,189 per year at home €91,798 per year in hospital enditemize	Cost of IVIG given at home vs. in hospital
Guptill/USA/2014 (17)	$108,016 per year	($9,720 per infusion)
Divino/USA/2018 (18)	$136,892 per year	51.2% of cost relates to cost of IVIG given in hospital
**HSCT**		
Burt/USA/2020	$108,577 (one time cost not per year)	Revenue collected $140,812

€, *euro; £, pound; $, dollar; CIDP, chronic inflammatory demyelinating polyradiculoneuropathy; IVIG, intravenous immunoglobulin; UK, United Kingdom; USA, United States of America*.

### Clinical Outcome: IVIG vs. HSCT

INCAT disability scale gives 5 points for upper and 5 for lower limb dysfunction with a total score of 10 that goes from 0 for normal to 10 for incapacitated upper and lower limbs. The ground-breaking ICE study of IVIG enrolled 59 treatment naïve CIDP patients with significant disability (mean INCAT score of 4.2) and demonstrated significant improvements after 24 weeks (4). During the second 24-week extension phase, patients remained stable (Table 2). The SCIg PATH study enrolled 59 treatment naïve of minimally treatment experienced (~1/3 of the study cohort received on average 4 courses IVIG over 9 months) patients with less severe disease (mean INCAT score of 2.0) (5). The PATH study demonstrated a significant treatment effect compared to continued decline on placebo, both with low (0.2 g/kg) or high (0.4 g/kg) SCIg dosing. However, when compared to base line, the mean INCAT scores did not improve (Table 2). For treatment of a chronic disease, both of these pivotal studies had relatively short follow-up of 48 and 24 weeks, respectively. Neither study reported full results of NCS although ICE reported no significant changes in CMAP compared to placebo. ICE reported no significant improvement in quality of life compared to placebo control (21).

**Table 2:** Comparison of changes in outcome parameters from baseline for ICE (IVIG) and PATH (SCIg) studies vs. hematopoietic stem cell transplantation (HSCT).

**Study (number of patients)**	**INCAT disability score**	**MRC sum score**	**Dominant Grip Kg**	**NCV (m/Sec)**	**CMAP (millivolts)**	**SF-36 QOL physical/mental**
**IVIG studies for naïve or minimally IVIG treated (*****<*****4 infusion in 9 months) CIDP**						
*ICE Study baseline (N = 59) (reference 4)*	4.2	49.3	48.2	NR	NR	31.1/46.3
ICE 1st 24 weeks of IVIG change from baseline	−1.1	+3.3	+13.2	NR	NS	+5.7/+3.3
ICE 2nd 24 weeks of IVIG change from baseline	0.1	0.8	−0.8	NR	NR	NR
*PATH Study low dose baseline (N = 57) (reference 5)*	2.0	75	67	NR	NR	NR
PATH low-dose SCIg 24 week change from baseline	0	0	−0.6	NR	NR	NR
*PATH study high dose baseline (N = 58) (reference 5)*	2.0	76	68.4	NR	NR	NR
PATH High-dose SCIg 24 week change from baseline	0	0	−2.7	NR	NR	NR
**HSCT for chronic IVIG dependent CIDP (*****n*** **=** **60) (reference 16)**						
Baseline value	4.4	51.8	18	22.7	3.55	29.3/47.7
6 month	−1.3	+3.0	+4.7	+8.8	+0.14	+19.5/+16.7
1 year	−2.3	+5.2	+8.3	+10.8	+1.08	+28.3/+19.7
2 years	−2.3	+5.2	+11.2	+11.1	+1.03	+27.9/+21
3 years	−2.3	+5.2	+10.8	+15	+1.69	+33.4/+25.4
4 years	−2.4	+5.2	+12.4	+15.4	+1.72	+30.3/+22.8
5 years	−2.6	+5.2	+12.8	+15.6	+0.57	+36.6/+33

*CMAP, compound motor action potential; ICE, Intravenous immune globulin for chronic inflammatory demyelinating polyradiculoneuropathy; HSCT, hematopoietic stem cell transplantation; INCAT, inflammatory neuropathy cause and treatment; IVIG, intravenous immunoglobulin; MRC, medical research council; NCV, nerve conduction velocity; NR, not reported; NS, not significant; QOL, quality of life; PATH, Polyneuropathy And Treatment with Hizentra; SCIg, subcutaneous immunoglobulin; SF-36, short form 36. From ICE study (4) and PATH study (5)*.

**Figure 2:**
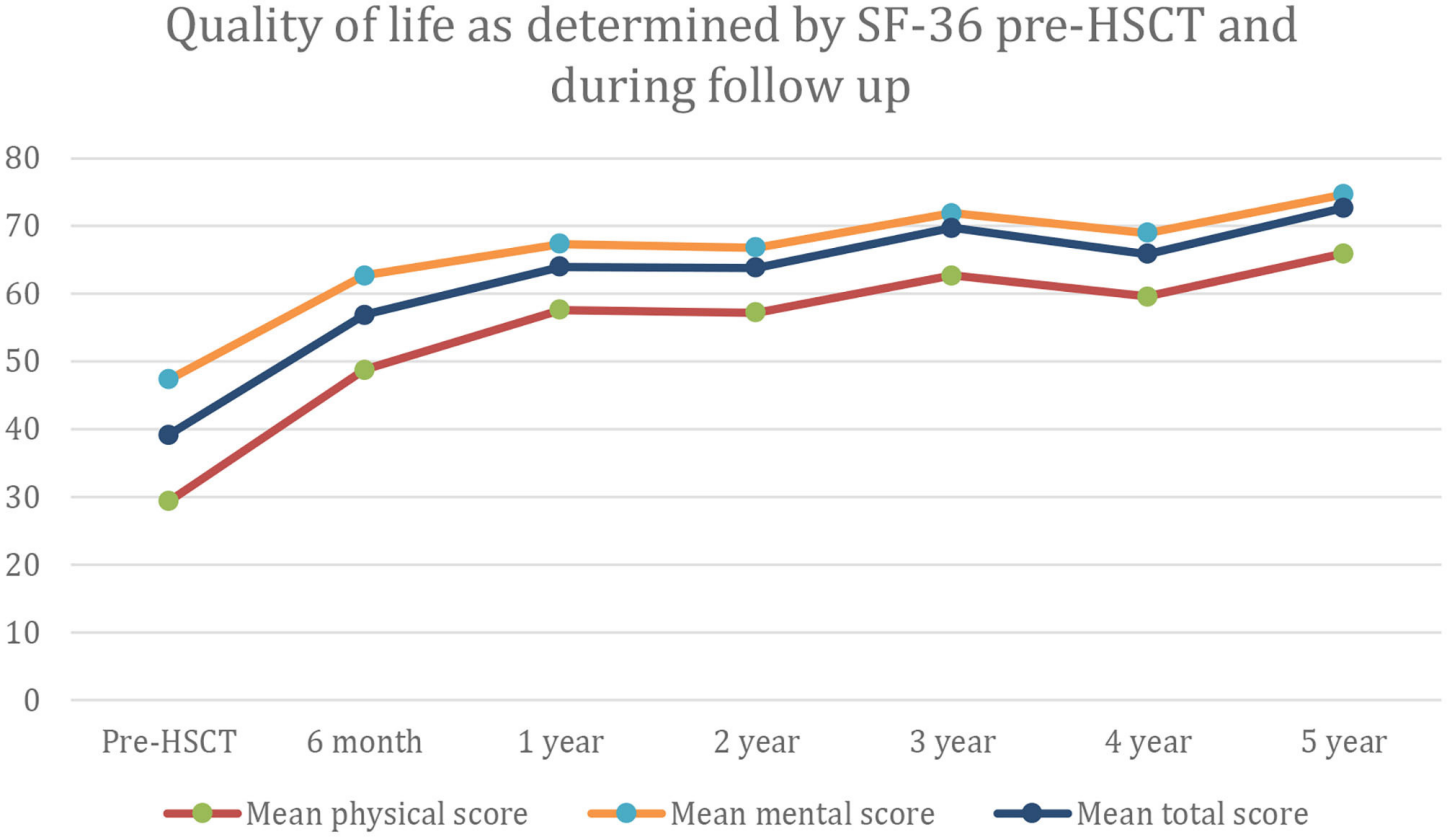
Quality of life as measured by the SF-36 rating scale at baseline and for up to 5 years after hematopoietic stem cell transplantation (HCST).

**Figure 3:**
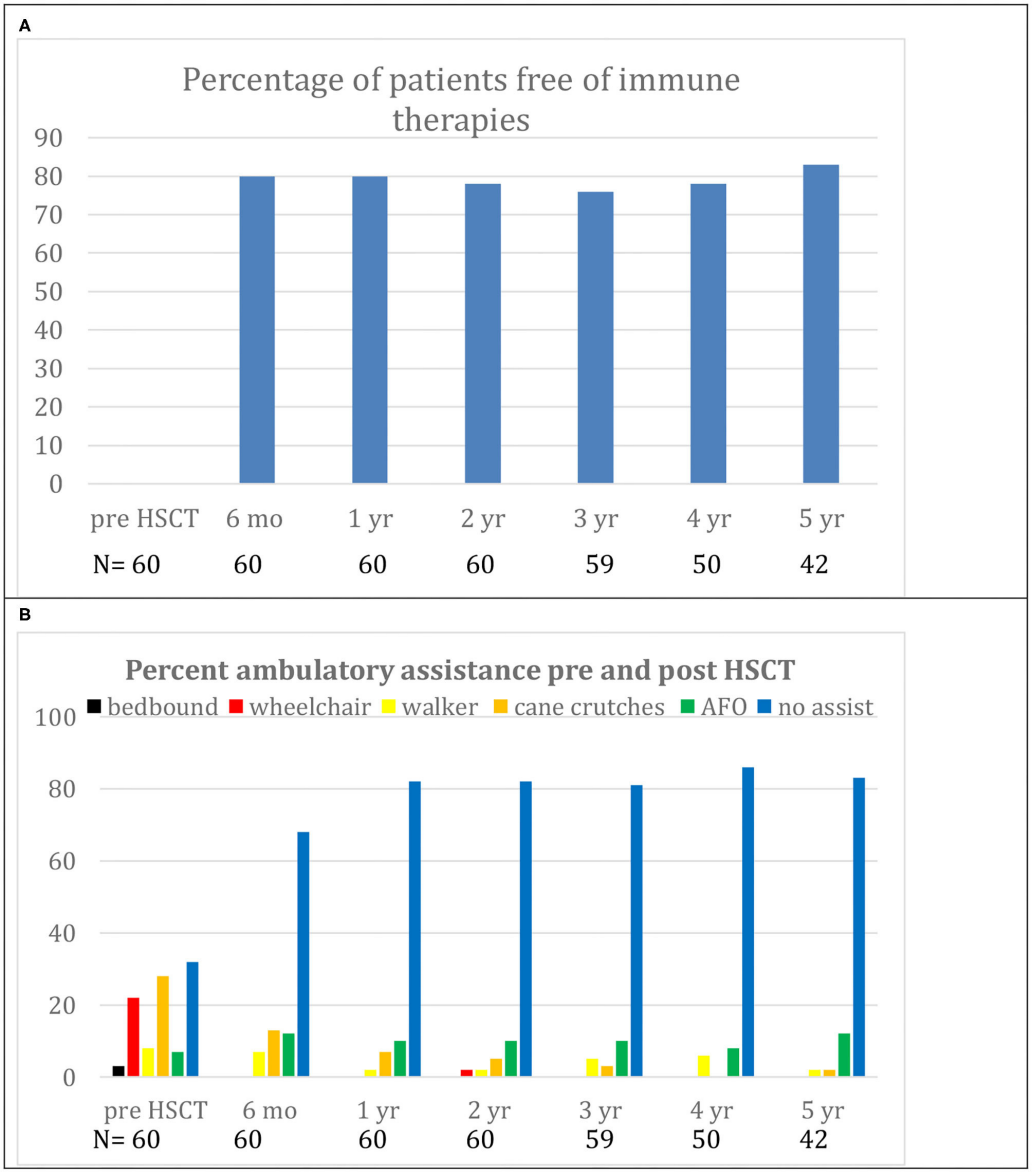
**(A)** Demonstrates freedom from immunological therapies for up to 5 years after hematopoietic stem cell transplantation (HSCT). **(B)** Demonstrates reduction in use of ambulatory aids for up to 5 years after HSCT. Replicated with permission from source (16).

In the only prospective autologous HSCT trial for CIDP, 60 patients were selected for being IVIG dependent with a mean treatment duration of 6 years and a significant chronic baseline disability (mean INCAT score of 4.4) (16). Following a one-time treatment with autologous HSCT, patients had significant improvements in INCAT score, QOL, and both NCV and CMAP that persisted for 5 years (Table 2, Figure 2). During a 5-year interval after HSCT, all patients demonstrated marked improvements in unassisted ambulation and 80% of patients remained free of all immune-based therapies including IVIG (Figure 3) (16).

## Discussion

By convention, whenever, hematopoietic stem cells are infused after chemotherapy, the procedure is called a hematopoietic stem cell transplant. In reality, there is no transplant of foreign tissue or transplant of autologous tissue to a heterologous location. The unmanipulated hematopoietic stem cells have no disease-specific or disease-modulating effects. In fact, since the conditioning regimen herein is non-myeloablative, hematopoietic recovery will occur without infusion of previously collected autologous hematopoietic stem cells. Thus, the hematopoietic stem cells are in reality an autologous supportive blood product transfused to shorten the period of chemotherapy-induced cytopenias, i.e., anemia, thrombocytopenia, and neutropenia, similar to the rationale for infusion of platelets or red blood cells after chemotherapy.

HSCT, unlike immune suppressive pharmacology drugs, is given as a once-only treatment. The mechanism of autologous HSCT is based on the rationale that removal of lymphocytes with a short course of chemotherapy/biologics (6 days) and subsequent regeneration of hematopoiesis over 9–10 days occurring in the absence of cytokine inflammatory signals will result in a return of tolerance toward self-epitopes and self-tissue. In order to confirm this hypothesis, immune analysis before and after HSCT for autoimmune disorders is an area of active research. Autologous non-myeloablative HSCT for multiple sclerosis, an immune-mediated central nervous system demyelinating disease, resulted in an increased diversity and normalization of the T cell receptor repertoire consistent with “out with the old and in with the new” (22, 23). After HSCT, there is also an increase in recent thymic emigrants and suppressor T regulatory cells (Treg) (24–26) consistent with re-establishment of tolerance.

The mean per CIDP patient annual IVIG treatment costs in the USA were reported to be $108,016 and $136,892 in 2014 and 2018, respectively (17, 18) which is approximately equal to the cost of HSCT (cost $108,577, revenue collected $140,812). Since HSCT is a one-time treatment and 80% of patient remain treatment-free for over 5 years after HSCT (16), the projected health care savings per patient over that 5-year interval would be $438,054 ($136,892 × 4 × 0.8). However, this estimate may be an under-estimate since it assumes that IVIG prices will not increase over that 5-year interval and is based on health care costs for the average CIDP patient.

The patients treated with HSCT were not representative of the overall CIDP population as they did not remit with chronic IVIG therapy (average 6 years) and the dosages of IVIG were higher than the average patient. The immediate pre-HSCT mean monthly dose of IVIG was 151 grams in this cohort of patients. For a seventy-kilogram person infused at 500 mg/kg, each infusion would be 35 grams. Thus, to reach a mean IVIG dose 151 grams per month, each person on average would receive 4.3 infusions a month. Since the average cost per IVIG infusion in the USA has been reported to be $9,720, and patients on average received 4.3 infusions per month, the IVIG costs would be $41,796 per month. In this subset of patients, the revenue collected from HSCT ($140,812) would therefore pay for itself after stopping 4 months of conventional IVIG treatment ($140,812/41,796).

There are several limitations of this study. The cost for HSCT is from one center while costs for HSCT would likely be affected by agents used in the conditioning regimen, whether the regimen is myeloablative or non-myeloabaltive, center experience, and national and international regional variation. For example, in the United Kingdom (UK) National Health Service (NHS) the cost of HSCT is approximately £30,000–35,000 compared to an annual UK cost for IVIG of £49,430 (12, 13). While costs for HSCT in the UK public health system appears cheaper, in public health systems, the overhead costs are not included in patient costs. In contrast, in the American private health care system both direct costs and overhead costs are included because both must be recovered in the patient billing. Furthermore, while patients stopped and remained off IVIG and other immune based treatments after HSCT, other post-transplant costs, e.g., blood monitoring, are not included. Outpatient pharmaceutical IVIG rebates are proprietary information that could not be factored into this analysis. A long-term parameter affecting cost effectiveness is risk of late malignancies. None of the patients undergoing HSCT with the less intense non-myeloablative regimen developed a malignancy. However, the risk of malignancy will depend on the conditioning regimen, will take long-term (>10–20 years) follow-up, and will likely vary between regimens with the more intense myeloablative regimens containing multiple high dose alkylating agents and or irradiation having a higher risk. Finally, this analysis does not factor in loss of work productivity and wages from CIDP related disability which due to the superior health outcome and reversal of neurologic disability after HSCT should favor HSCT compared to continuing IVIG.

Finally, immunoglobulin availability and costs vary widely depending on the product (https://bnf.nice.org.uk/medicinal-forms/normal-immunoglobulin.html), with manufacturers ranging from pharmaceutical companies to transfusion services. Moreover, irrespective of cost, there are important considerations relating to availability of a non-renewable limited supply of immunoglobulins as a pooled human blood product. In view of this, some health services, such as the UK NHS, enforce demand management schemes whereby administration and expenditure can be monitored and regulated (http://igd.mdsas.com/).

As costs will differ between health services, manufacturers of immunoglobulins (pharmaceutical or transfusion services) and providers of HSCT, further studies are required to determine cost-effectiveness of HSCT across local settings. Independent of costs, there are also important considerations related to supply of immunoglobulins that are derived from a finite quantity of pooled human blood products.

## Summary

This is the first report of comparison of costs and health outcomes for HSCT compared to chronic IVIG that, over a 5-year interval and in properly selected patients, appears to result in a health care savings of ~500,000.00 US dollars per patient. It appears that when using a non-myeloablative regimen in properly selected patients that HSCT may be performed safely, could achieve a substantial cost savings for private or public insurance, and give superior results such as improvement in NCS and QOL compared to continued IVIG. However, further investigation and analysis are needed and appear to be warranted.

## Data Availability Statement

The raw data supporting the conclusions of this article will be made available by the authors, contingent upon hospital approval.

## Ethics Statement

The study involving clinical outcome of human participants was reviewed and approved by Institutional Review Board. The patients/participants provided their written informed consent before treatment.

## Author Contributions

All authors listed have made a substantial, direct and intellectual contribution to the work, and approved it for publication.

## Conflict of Interest

The authors declare that the research was conducted in the absence of any commercial or financial relationships that could be construed as a potential conflict of interest.

## References

[1] Van den Bergh PY Hadden RD Bouche P Cornblath DR Hahn A Illa I. European Federation of Neurological Societies/Peripheral Nerve Society guideline on management of chronic inflammatory demyelinating polyradiculoneuropathy: report of a joint task force of the European Federation of Neurological Societies and the Peripheral Nerve Society—first revision. Eur J Neurol. (2010) 17:356–63. 10.1111/j.1468-1331.2009.02930.x 20456730

[2] Peltier AC Donofrio PD. Chronic inflammatory demyelinating polyradiculoneuropathy: from bench to bedside. Semin Neurol. (2012) 32:187–95. 10.1055/s-0032-1329194 23117943 PMC4845954

[3] Mendell JR Barohn RJ Freimer ML Kissel JT King W Nagaraja HN. Randomized controlled trial of IVIg in untreated chronic inflammatory demyelinating polyradiculoneuropathy. Neurology. (2001) 56:445–9. 10.1212/WNL.56.4.445 11222785

[4] Hughes RA Donofrio P Bril V Dalakas MC Deng C Hanna K. Intravenous immune globulin (10% caprylate-chromatography purified) for the treatment of chronic inflammatory demyelinating polyradiculoneuropathy (ICE study): a randomised placebo-controlled trial. Lancet Neurol. (2008) 7:136–44. 10.1016/S1474-4422(07)70329-0 18178525

[5] Van Schaik IN Bril V van Geloven N Hartung HP Lewis RA Sobue G. Subcutaneous immunoglobulin for maintenance treatment in chronic inflammatory demyelinating polyneuropathy (PATH): a randomized, double blinded, placebo controlled, phase 3 trial. Lancet Neurol. (2018) 17:35–46. 10.1186/s13063-016-1466-2 29122523

[6] Dyck PJ Daube J O'Brien P Pineda A Low PA Windebank AJ. Plasma exchange in chronic inflammatory demyelinating polyradiculoneuropathy. N Engl J Med. (1986) 314:461–5. 10.1056/NEJM198602203140801 3511382

[7] Gorson KC Allam G Ropper AH. Chronic inflammatory demyelinating polyneuropathy: clinical features and response to treatment in 67 consecutive patients with and without monoclonal gammopathy. Neurology. (1997) 48:321–8. 10.1212/WNL.48.2.321 9040714

[8] Kuitwaard K van Doorn PA. Newer therapeutic options for chronic inflammatory demyelinating polyradiculoneuropathy. Drugs. (2009) 29:987–1001. 10.2165/00003495-200969080-00004 19496628

[9] Rabin M Mutlu G Stojkovic T Maisonobe T Lenglet T Fournier E. Chronic inflammatory demyelinating polyradiculoneuropathy: search for factors associated with treatment dependence or successful withdrawal. J Neurol Neurosurg Psychiatry. (2014) 85:901–6. 10.1136/jnnp-2013-306105 24309269

[10] Querol L Rojas-Garcia R Casasnovas C Sedano MJ Muñoz-Blanco JL Alberti MA. Long-term outcome in chronic inflammatory demyelinating polyneuropathy patients treated with intravenous immunoglobulin: a retrospective study. Muscle Nerve. (2013) 48:870–6. 10.1002/mus.23843 23512566

[11] Cocito D Paolasso I Antonini G Benedetti L Briani C Comi C. A nationwide retrospective analysis on the effect of immune therapies in patients with chronic inflammatory demyelinating polyradiculoneuropathy. Eur J Neurol Févr. (2010) 17:289–94. 10.1111/j.1468-1331.2009.02802.x 19863650

[12] Mahdi-Rogers M McCrone P Hughes RA. Economic costs and quality of life in chronic inflammatory neuropathies in south- east England. Eur J Neurol. (2014) 21:34–39. 10.1111/ene.12245 23930744

[13] Rajabally YA Afzal S. Clinical and economic comparison of an individualised immunoglobulin protocol vs. standard dosing for chronic inflammatory demyelinating polyneuropathy. J Neurol. (2019) 266:461–7. 10.1007/s00415-018-9157-4 30556098 PMC6373347

[14] Robertson EE Donofrio PD. Treatment of chronic inflammatory demyelinating polyneuropathy. Curr Treat Options Neurol. (2010) 12:84–94. 10.1007/s11940-010-0058-9 20842572

[15] Blackhouse G Gaebel K Xie F Campbell K Assasi N Tarride JE. Cost-utility of intravenous immunoglobulin (IVIG) compared with corticosteroids for the treatment of chronic inflammatory demyelinating polyneuropathy (CIDP) in Canada. Cost Eff Resour Alloc. (2010) 8:14. 10.1186/1478-7547-8-14 20565778 PMC2903512

[16] Burt RK Balabanov R Tavee J Han X Sufit R Ajroud-Driss S. Hematopoietic stem cell transplantation for chronic inflammatory demyelinating polyradiculoneuropathy. J Neurol. (2020) 267:3378–91. 10.1007/s00415-020-10010-6 32594300

[17] Guptill JT Bromberg MB Zhu L Sharma BK Thompson AR Krueger A. Patient demographics and health plan paid costs in chronic inflammatory demyelinating polyneuropathy. Muscle Nerve. (2014) 50:47–51. 10.1002/mus.24109 24639235

[18] Divino V Mallick R DeKoven M Krishnarajah G. The economic burden of CIDP in the United States: a case-control study. PLoS ONE. (2018) 13:e0206205. 10.1371/journal.pone.0206205 30352101 PMC6198979

[19] Le Masson G Solé G Desnuelle C Delmont E Gauthier-Darnis M Puget S. Home versus hospital immunoglobulin treatment for autoimmune neuropathies: a cost minimization analysis. Brain Behav. (2018) 8:e00923. 10.1002/brb3.923 29484273 PMC5822576

[20] Mengel D Fraune L Sommer N Stettner M Reese JP Dams J. Costs of illness in chronic inflammatory demyelinating polyneuropathy in Germany. Muscle Nerve. (2018) 58:681–687. 10.1002/mus.26315 30073683

[21] Merkies IS Bril V Dalakas MC Deng C Donofrio P Hanna K. Health-related quality-of-life improvements in CIDP with immune globulin IV 10%: the ICE Study. Neurology. (2009) 72:1337–44. 10.1212/WNL.0b013e3181a0fd80 19365055

[22] Muraro PA Douek DC Packer A Chung K Guenaga FJ Cassiani-Ingoni R. Thymic output generates a new and diverse TCR repertoire after autologous stem cell transplantation in multiple sclerosis patients. J Exp Med. (2005) 201:805–16. 10.1084/jem.20041679 15738052 PMC2212822

[23] Van Epps HL. Out with the old, in with the new. J Exp Med. (2005) 201:663–4. 10.1084/jem2015iti318997218

[24] Arruda LCM de Azevedo JTC de Oliveira GLV Scortegagna GT Rodrigues ES Palma PVB. Immunological correlates of favorable long-term clinical outcome in multiple sclerosis patients after autologous hematopoietic stem cell transplantation. Clin Immunol. (2016) 169:47–57. 10.1016/j.clim.2016.06.005 27318116

[25] Karnell FG Lin D Motley S Duhen T Lim N Campbell DJ. Reconstitution of immune cell populations in multiple sclerosis patients after autologous stem cell transplantation. Clin Exp Immunol. (2017) 189:268–27. 10.1111/cei.12985 28498568 PMC5543487

[26] Abrahamsson SV Angelini DF Dubinsky AN Morel E Oh U Jones JL. Non-myeloablative autologous haematopoietic stem cell transplantation expands regulatory cells and depletes IL-17 producing mucosal-associated invariant T cells in multiple sclerosis. Brain J Neurol. (2013) 136 (Pt 9):2888–903. 10.1093/brain/awt182 23864273 PMC3754461

